# Acupuncture and related therapies for atopic eczema: A protocol for systematic review and network meta-analysis

**DOI:** 10.1097/MD.0000000000031956

**Published:** 2022-12-16

**Authors:** Wang Li, Kaiqi Zhang, Dongxin Wang, Ruimin Jiao, Sha Li, Xu Zhai

**Affiliations:** a Graduate School, China Academy of Chinese Medical Sciences, Beijing, China; b Institute of Acupuncture and Moxibustion, China Academy of Chinese Medical Sciences, Beijing, China; c Wangjing Hospital of China Academy of Chinese Medical Sciences, Beijing, China; d Blood collection center, Qingdao Women and Children’s Hospital, Qingdao, Shandong, China.

**Keywords:** acupuncture, atopic eczema, network meta-analysis, systematic review

## Abstract

Atopic eczema (AE) is a chronic relapsing dermatological disease characterized by pruritus, dryness, erythema, and lichenification. Acupuncture as an alternative treatment can relieve symptoms, reduce the recurrence, and improve the quality of life (QoL) with less adverse reactions in treating AE. This network meta-analysis (NMA) will **c**omprehensively access the efficacy and safety of different acupuncture therapies for patients with AE, and rank the different acupuncture therapies, in order to present evidence-based medicine evidence for clinical application extensively.

**Method and analysis:**

This study will search 8 electronic databases from the establishment of the database to August 30th, 2022. The screening of literature, data extraction, and risk of bias assessment will be conducted by 2 researchers, respectively. The quality of evidence will be judged by the Grading of Recommendations Assessment, Development and Evaluation system. This NMA will be analyzed with Stata Version. 14.0 and WinBUGS Version.1.4.3.

**Results:**

This study will comprehensively access the efficacy and safety of different acupuncture therapies for patients with AE on the severity, itch intensity, emotional symptoms, QoL, and recurrence rate. Moreover, it will further identify which acupuncture therapy is the most effective.

**Conclusion:**

The findings of this NMA may help patients and therapists choose the best acupuncture therapy in treating AE and furnish reliable evidence for guidelines.

**Registration number:**

PROSPERO CRD42020203437

## 1. Introduction

Atopic eczema (AE), is a chronic relapsing dermatological disease characterized by pruritus, skin dryness, erythema, and lichenification.^[1]^ Epidemiological studies have shown that AE impacts approximately 15% to 20% of children and 1% to 3% of the general population worldwide, and it also affects about 7.5% of Chinese. The economic burden of direct and indirect costs is about 37.7 billion dollars of extra expenses, which are shared by families and nursing staff.^[2]^

Topical corticosteroids, antihistamines, and calcineurin inhibitor are mainly used for the first-line medication for AE.^[3,4]^ However, these medications have their limitations, such as, skin local irritation, burning, and even skin infection by virus, and these could cause resistance and dependence of these drugs as well.^[3,5]^

Acupuncture is considered to be an alternative treatment for AE, which can relieve symptoms, reduce the recurrence, and improve the quality of life (QoL) with less adverse reactions.^[6]^ Moreover, there are various kinds of acupuncture therapies, which may or may not penetrate specific acupoints, such as manual acupuncture (MA), electroacupuncture (EA), acupressure, and etc.^[7]^ As a part of traditional Chinese medicine, acupuncture has been used to treat various diseases, including skin diseases.^[7]^ Previous systematic review have also concluded that acupuncture is an effective and safe treatment for AE. And there are several randomized controlled trials (RCTs) which have reported different techniques of acupuncture may relieve symptoms of AE.^[8]^ Results from animal studies suggest that high frequency EA can release dynorphin to relieve the itching of AE rats induced by capsaicin-injection.^[9]^ And the LI-11 (QC) acupoint blocks serotonin 5-H2 and 5-H7 receptors probably for alleviating skin inflammation and pruritus evoked by serotonergic,^[10]^ it also inhibits the serum IgE levels and the releasing of proinflammatory cytokines and proteins such as IL-4, IL-8, NF-κB, JNK in treating AE.^[11]^

So far, patients with AE have adopted different methods of acupuncture in previous studies.^[8]^ However, these different acupuncture therapies have no comprehensive comparison between each other. Thus, this study aims to use the network meta-analysis (NMA) method^[12]^ to evaluate the effectiveness and safety of different acupuncture therapies in patients with AE, and identify which acupuncture therapy is the most effective by integrating all available evidence.

## 2. Methods

### 2.1. Study registration

This protocol of NMA has been registered in the website of PROSPERO (https://www.crd.york.ac.uk/prospero/display_record.php?RecordID=203437). Moreover, this protocol of NMA has been complianced with reporting statements of the Preferred Reporting Items for Systematic Reviews and Meta-Analyses protocol (PRISMA-P) checklist.^[13]^

### 2.2. Eligibility criteria

#### 2.2.1. Type of included studies.

RCTs in English or Chinese will be included for analysis.

#### 2.2.2. Participants.

Patients will include those diagnosed with AE^[1]^ regardless of age, gender, or race.

#### 2.2.3. Intervention.

##### 2.2.3.1. Definition of acupuncture.

Acupuncture therapies are defined according to the World Health Organization^[14]^: Acupuncture literally means to acupuncture with a needle. Moreover, this study may also include the applying other types of stimulation to acupoints. The study contains common acupuncture modalities (percutaneous acupuncture and noninvasive methods), including MA, EA, transcutaneous electrical nerve stimulation of acupoint, laser acupuncture, acupressure, scalp acupuncture, ear (auricular) acupuncture, heat lamps and magnets with no limitation of intensity, doses, administration, or any personnel administering of acupuncture.

#### 2.2.4. Comparisons.

The control group will include no-treatment, sham acupuncture, waiting list, or the same conventional medical treatment in the intervention group.

#### 2.2.5. Outcomes.

The primary outcomes will contain the eczema area and severity index (EASI),^[15]^ the severity scoring of atopic dermatitis (SCORAD),^[16]^ and the itch intensity measured by visual analogue scale (VAS).^[17]^

Secondary outcomes contain global symptom improvement based on the score of EASI, QoL, recurrence rate and adverse events.

### 2.3. Data sources and search strategy

This study will search 8 electronic databases, including PubMed, EMBASE, Cochrane Database of Systematic Review, Cochrane Central Register of Controlled Trials, China National Knowledge Infrastructure, Wanfang Database, Chinese Biomedical Literature Database, and Chinese Scientific Journal Database, from their establishment to August 30th, 2022. This study will combine medical subject heading terms and free terms as search term, what can be categorized into 3 parts: clinical condition (e.g., “atopic eczema,” “dermatitis, atopic,” “atopic dermatitides,” “atopic dermatitis”), intervention (e.g., “acupuncture,” “electroacupuncture,” “auricular needle,” “scalp Stimulation”) and study design (e.g., “randomized controlled trial,” “clinical trial”). We will adjust the search terms according to the retrieval strategy of each database (Search strategy in 8 databases is shown in Supplementary 1, http://links.lww.com/MD/H997).

### 2.4. Study selection and data extraction

**Figure 1.**
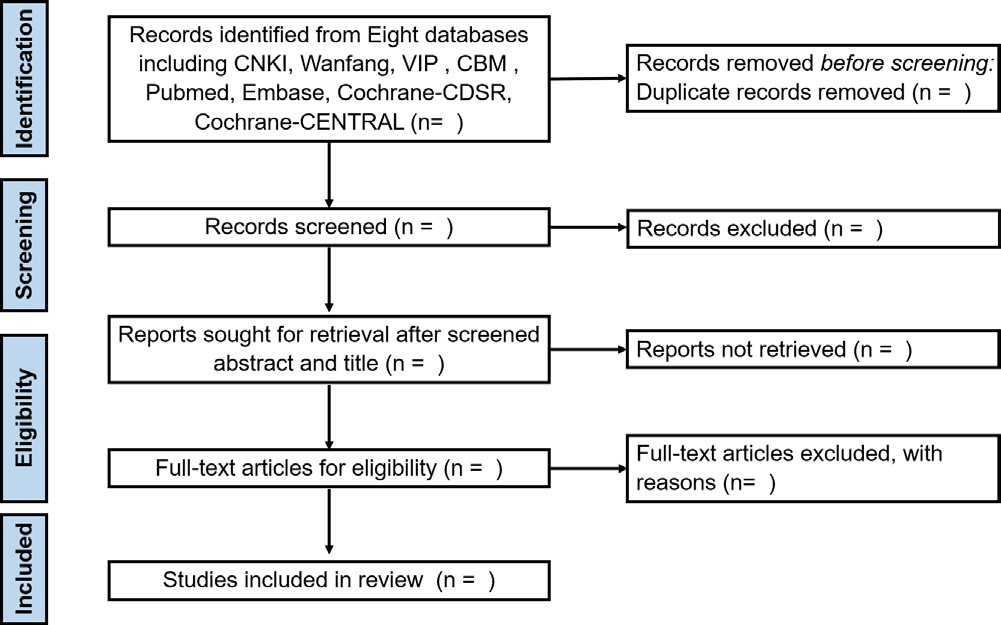
Flow chart of study inclusion.

Two researchers (DW and KZ) will independently screen the studies according to the selection criteria and conduct the data extraction. Any disagreements will be discussed and resolved by the 2 researchers. If no agreement is made, a third researcher (XZ) will resolve it through arbitration. If the literature only published with abstracts, researchers will contact and get full-text or more contents. The PRISMA flow chart of the selection process is displayed in Figure 1.

These data will be extracted and formed the table as follows: title, authors’ names, publication date, sample size, methods of randomization, allocation concealment, and blinding, comparisons, intervention, and outcomes. The data extraction and the management of all references will be conducted respectively by Microsoft Excel 2016 and EndNote X9.

### 2.5. Risk of bias assessment

Two independent researchers (WL and SL) will use the Cochrane Collaboration Risk of Bias Tool^[18]^ to access the risk of bias of the included trials. Any disagreements will be discussed and resolved by the 2 researchers. If no agreement is made, a third researcher (XZ) will resolve it through arbitration.

### 2.6. Summary of evidence

Two independent researchers (WL and RJ) will use the Grading of Recommendations Assessment, Development and Evaluation (GRADE) system to access the overall of evidence.^[19]^ Any disagreements will be discussed and resolved by the 2 researchers. If no agreement is made, a third researcher (XZ) will resolve it through arbitration. The evaluation of the GRADE system includes risk of bias, indirectness, inconsistency, inaccuracy and publication bias, and it will be ranked as very low, low, moderate and high.

### 2.7. Data synthesis and analysis

Continuous data will use the mean difference (MD) and 95% credible intervals (CIs), and dichotomous data will use the odds ratio (OR) and 95% CIs. The heterogeneity across studies will be explored by using the *I^2^* statistic and p values. *I^2 ^*> 50% indicates significant heterogeneity, and *P* < .01 exposes considerable heterogeneity. NMA will be performed to simultaneously compare multiple interventions. The network statistical analysis will be performed by WinBUGS Version.1.4.3 with a random effects model for direct and indirect comparison. And the Bayesian Markov chain Monte Carlo methods will be adopted to calculate. The number of iterations will be set to 50000 (the 1000 iterations for the annealing algorithm and the 10001 iterations for the sampling). The surface under the cumulative ranking curve (SUCRA) analysis in Stata Version. 14.0 will be used to rank the effect of treatments of all included interventions. Moreover, node-splitting method and loop-specific approach will be adopted to evaluate the inconsistency between direct and indirect evidence.^[20,21]^ We will use a consistency model if *P* value is >.05, which indicates a better consistency between direct and indirect comparisons. If *P* value is <.05, we will use the inconsistency model. Moreover, if node split does not generate, we will use the consistency model to analyze.

### 2.8. Sensitivity analysis

The sensitivity analysis will explore the robustness of the pooled outcomes in accordance with size of sample, method of acupuncture therapy, or type of comparisons. We will delete each study from this NMA one by one and recalculate the summary effect. If the removal of this substance will change the size of the mixing effect by > 10%, the study will be considered influential.^[22]^

### 2.9. Publication bias

The funnel plots will explore the publication bias of this study, and Egger’s test will be used to evaluate its asymmetry. Additionally, this NMA will also explore the publication bias further using appropriate network meta-regression and models.

## 3. Discussion

AE is an occupational skin disease of adult, which may commonly lead to the development of other atopic diseases like rhinitis and/or asthma.^[22]^ Doctors and administrators have recognized that AE is a major handicap with a considerable personal, social, and financial burden on the family for years.^[23]^

Although many guidelines of AE suggest using pharmacological therapy for treating AE, it may cause unsatisfactory therapeutic effect or terminating of treatment due to adverse reactions or frequently recrudesce for many patients with AE.^[1]^ Since recent years, diverse acupuncture and related therapies have been widely used during the process of the clinical practice for patients with AE, moreover, the integrated 2 or more acupuncture therapies have been used in several trials.^[8,24,25]^ Thus, this study is the first using NMA on acupuncture for AE with integrating the most comprehensive data. This NMA will also use the Bayesian statistical methods to measure the comparable probability for ranking many different acupuncture therapies and then report the ranking. In addition, some limitations such as low-quality trials, or heterogeneity of different studies have still existed in this NMA, which will may affect the final results. In summary, ranking acupuncture therapies for patients with AE will be expected in this NMA through comparing the efficacy and safety of acupuncture. This study may help patients and therapists choose the best acupuncture therapy in treating AE and furnish reliable evidence for guidelines.

## Author contributions

**Conceptualization:** Xu Zhai, Wang Li, Ruimin Jiao.

**Data curation:** Kaiqi Zhang, Sha Li.

**Formal analysis:** Kaiqi Zhang, Sha Li.

**Funding acquisition:** Xu Zhai.

**Investigation:** Xu Zhai.

**Methodology:** Wang Li, Ruimin Jiao, Dongxin Wang.

**Resources:** Ruimin Jiao, Sha Li.

**Software:** Kaiqi Zhang, Sha Li.

**Supervision:** Xu Zhai.

**Writing – original draft:** Wang Li, Kaiqi Zhang, Ruimin Jiao.

**Writing – review & editing:** Xu Zhai, Wang Li, Kaiqi Zhang, Ruimin Jiao.

## Supplementary Material
