# Supplementary material for: Acupuncture and related therapies for atopic eczema: A protocol for systematic review and network meta-analysis
Source: Medicine (Baltimore). 2022 Dec 16;101(50):e31956. doi: 10.1097/MD.0000000000031956 (PMC9771330; doi:10.1097/MD.0000000000031956)
Supplement: Supplementary file 1 [file medi-101-e31956-s001.pdf]

Supplementary 1 search strategy for databases

Table 1 Search strategy for Pubmed (Website: https://www.ncbi.nlm.nih gov/pubmed)

Number	Search terms
#1	randomized controlled trial[Publication Type] OR controlled clinical trial[Publication Type] OR clinical trial[Publication Type]) OR (Clinical Trials as Topic[MeSH Terms] OR Randomized Controlled Trials as Topic[MeSH Terms] OR Controlled Clinical Trials as Topic[MeSH Terms] OR Double-Blind Method[MeSH Terms] OR single-blind method[MeSH Terms] OR Control Groups[MeSH Terms] OR Random Allocation[MeSH Terms] OR cross-over studies[MeSH Terms] OR drug therapy[MeSH Subheading])) OR (randomized[Title/Abstract] OR randomization[Title/Abstract] OR placebo[Title/Abstract] OR randomly[Title/Abstract] OR trial[Title/Abstract] OR groups[Title/Abstract] OR crossover[Title/Abstract] OR cross-over[Title/Abstract]
#2	"atopic eczema" [Mesh] OR "Dermatitis, Atopic" [Mesh]) OR ("Atopic Dermatitides" [Title/Abstract] OR "Atopic Dermatitis" [Title/Abstract] OR "Dermatitides, Atopic" [Title/Abstract] OR "Neurodermatitis, Atopic" [Title/Abstract] OR "Atopic Neurodermatitides" [Title/Abstract] OR "Atopic Neurodermatitis" [Title/Abstract] OR "Neurodermatitides, Atopic" [Title/Abstract] OR "Neurodermatitis, Disseminated" [Title/Abstract] OR "Disseminated Neurodermatitides" [Title/Abstract] OR "Disseminated Neurodermatitis" [Title/Abstract] OR "Neurodermatitides, Disseminated" [Title/Abstract] OR "Eczema, Atopic" [Title/Abstract] OR "Atopic Eczema" [Title/Abstract] OR "Eczema, Infantile" [Title/Abstract] OR "Infantile Eczema"
#3	Acupuncture[Title/Abstract]) OR Acupuncture[MeSH Terms]) OR Electroacupuncture[Title/Abstract]) OR Electroacupuncture[MeSH Terms]) OR Transcutaneous Electric Nerve Stimulation[Title/Abstract]) OR Transcutaneous Electric Nerve Stimulation[MeSH Terms]) OR auricular needle[Title/Abstract]) OR auricular needling[Title/Abstract]) OR Scalp Sensory[Title/Abstract]) OR Scalp Stimulation[Title/Abstract]) OR Filliform Needle[Title/Abstract]) OR endothelial needle[Title/Abstract]) OR Three Edged Needle[Title/Abstract]) OR Plum-blossom Needle[Title/Abstract]) OR Cutaneous Needle[Title/Abstract]) OR Fire Needle[Title/Abstract]) OR Cauterization With Heated Needle[Title/Abstract]) OR Auricular Plastertherapy[Title/Abstract]) OR Auricular Point Sticking[Title/Abstract]) OR Dry Needling [Title/Abstract]) OR Dry Needling[MeSH Terms]) OR Acupuncture, Ear[MeSH Terms]
#4	#1 AND #2 AND #3 NOT ("Animals"[Mesh] NOT ("Humans"[Mesh] AND "Animals"[Mesh])

Table 2 Search strategy for Embase (Website: https://www.embase.com/)

Number	Search terms
#1	'acupuncture'/exp OR 'transcutaneous electric nerve stimulation'/exp OR 'electroacupuncture'/exp OR 'dry needling'/exp OR 'acupuncture, ear'/exp
#2	acupuncture:ab,ti OR electroacupuncture:ab,ti OR 'transcutaneous electric nerve stimulation':ab,ti OR tens:ab,ti OR 'auricular needle':ab,ti OR 'auricular needling':ab,ti OR 'scalp sensory':ab,ti OR 'scalp stimulation':ab,ti OR 'filliform needle':ab,ti OR 'endothelial

	needle':ab,ti OR 'three edged needle':ab,ti OR 'plum-blossom needle':ab,ti OR 'cutaneous needle':ab,ti OR 'fire needle':ab,ti OR 'cauterization with heated needle':ab,ti OR 'auricular plastertherapy':ab,ti OR 'auricular point sticking':ab,ti OR 'dry needling':ab,ti
#3	'atopic dermatitides':ab,ti OR 'atopic dermatitis'':ab,ti OR 'dermatitides, atopic'':ab,ti OR tens:ab,ti OR 'neurodermatitis, atopic':ab,ti OR 'atopic neurodermatitides':ab,ti OR 'atopic neurodermatitis'':ab,ti OR 'neurodermatitides, atopic'':ab,ti OR 'neurodermatitis, disseminated''ab,ti OR 'disseminatedneurodermatitides':ab,ti OR 'disseminated neurodermatitis':ab,ti OR 'neurodermatitides, disseminated'':ab,ti OR 'eczema, atopic':ab,ti OR 'atopic eczema':ab,ti OR 'eczema, infantile':ab,ti OR 'infantile eczema':ab,ti
#4	#1 AND #2 AND #3 AND ([controlled clinical trial]/lim OR [randomized controlled trial]/lim)

Table 3 Search strategy for Cochrane (Website: https://www.cochranelibrary.com/)

Number	Search terms
#1	MeSh descriptor: [atopic dermatitides] explode all trees
#2	“atopic dermatitides”:ti,ab,kw OR “atopic dermatitis'':ti,ab,kwOR “dermatitides, atopic'':ti,ab,kw OR “neurodermatitis, atopic”:ti,ab,kw OR “atopic neurodermatitides”:ti,ab,kw OR “atopic neurodermatitis'':ti,ab,kw OR “neurodermatitides, atopic'':ti,ab,kw OR “neurodermatitis, disseminated'':ti,ab,kw OR “disseminated neurodermatitides”:ti,ab,kw OR “disseminated neurodermatitis”:ti,ab,kw OR “neurodermatitides, disseminated'':ti,ab,kw OR “eczema, atopic”:ti,ab,kw OR “atopic eczema”:ti,ab,kw OR “eczema, infantile”:ti,ab,kw OR “infantile eczema”:ti,ab,kw
#3	#1 OR #2
#4	MeSh descriptor: [acupuncture] explode all trees
#5	acupuncture:ti,ab,kw OR electroacupuncture:ti,ab,kw OR transcutaneous electric nerve stimulation:ti,ab,kw OR tens:ti,ab,kw OR auricular needle:ti,ab,kw OR auricular needling:ti,ab,kw OR scalp sensory:ti,ab,kw OR scalp stimulation:ti,ab,kw OR filliform needle:ti,ab,kw OR endothelial needle:ti,ab,kw OR three edged needle:ti,ab,kw OR plum-blossom needle:ti,ab,kw OR cutaneous needle:ti,ab,kw OR fire needle:ti,ab,kw OR cauterization with heated needle:ti,ab,kw OR auricular plastertherapy:ti,ab,kw OR auricular point sticking:ti,ab,kw OR dry needling:ti,ab,kw
#6	#4 OR #5
#7	#3 AND #6

Table 4 Search strategy for CNKI (Website: http:/www.cnki.net)

Search terms
(SU=(’针刺’+’电针’+’针灸’+’激光针’+’经皮电’+’经皮神经’+’电刺激’+’电止痛’+’体针’+’耳针’+’头针’+’毫针’+’干针’+’耳穴贴压’+’穴位按压’+’激光穴位照射’+’经皮电刺激治疗’+’经皮电刺激神经’+’经皮电刺激’+’针刺治疗’+’针灸疗法’+’经皮神经电刺激’+’激光穴位’-’动物’-’鼠’) OR TI=(’针刺’+’电针’+’针灸’+’激光针’+’经皮电’+’经皮神经’+’电刺激’+’电止痛’+’体针’+’耳针’+’头针’+’毫针’+’干针’+’耳穴贴压’+’穴位按压’+’激光穴位照射’+’经皮电刺激治疗’+’经皮电刺激神经’+’经皮电刺激’+’针刺治疗’+’针灸疗法’+’经皮神经电刺激’+’激光穴位’-’动物’-’鼠’) OR KY=(’针刺’+’电针’+’针灸’+’激光针’+’经皮电’+’经皮神经’+’电刺激’+’电止痛’+’体针’+’耳针’+’头针’+’毫针’+’干针’+’耳穴贴压’+’穴位

按压’+’激光穴位照射’+’经皮电刺激治疗’+’经皮电刺激神经’+’经皮电刺激’+’针刺治疗’+’针灸疗法’+’经皮神经电刺激’+’激光穴位’-’动物’-’鼠’） OR AB=(’针刺’+’电针’+’针灸’+’激光针’+’经皮电’+’经皮神经’+’电刺激’+’电止痛’+’体针’+’耳针’+’头针’+’毫针’+’干针’+’耳穴贴压’+’穴位按压’+’激光穴位照射’+’经皮电刺激治疗’+’经皮电刺激神经’+’经皮电刺激’+’针刺治疗’+’针灸疗法’+’经皮神经电刺激’+’激光穴位’-’动物’-’鼠’)) AND (SU=’随机’ or TI=’随机’ or KY=’随机’ or AB=’随机’) AND (SU=(’湿疹’+’湿疮’+’浸淫疮’+’旋耳疮’+’奶癣’+’乳头风’+’肾囊风’+’四弯风’+’脐疮’) OR TI=(’湿疹’+’湿疮’+’浸淫疮’+’旋耳疮’+’奶癣’+’乳头风’+’肾囊风’+’四弯风’+’脐疮’) OR KY=(’湿疹’+’湿疮’+’浸淫疮’+’旋耳疮’+’奶癣’+’乳头风’+’肾囊风’+’四弯风’+’脐疮’) OR AB=(’湿疹’+’湿疮’+’浸淫疮’+’旋耳疮’+’奶癣’+’乳头风’+’肾囊风’+’四弯风’+’脐疮’))

Table 5 Search strategy for Wanfang (Website: http://www.wanfangdata.com.cn/index.html)

Search terms
(((主题:”针刺” or 题名或关键词:”针刺”or 摘要:”针刺” or 主题:"电针" or 题名或关键词:"电针" or 摘要:"电针" or 主题:"激光针" or 题名或关键词:"激光针"or 摘要:"激光针" or 主题:"电刺激" or 题名或关键词:"电刺激" or 摘要:"电刺激" or 主题:"耳针" or 题名或关键词:"耳针"or 摘要:"耳针" or 主题:"耳廓针刺" or 题名或关键词:"耳廓针刺" or 摘要:"耳廓针刺" or 主题:"头针" or 题名或关键词:"头针" or 摘要:"头针" or 主题:"手针" or 题名或关键词:"手针" or 摘要:"手针" or 主题:"腹针" or 题名或关键词:"腹针"or 摘要:"腹针" or 主题:"毫针" or 题名或关键词:"毫针" or 摘要:"毫针" or 主题:"内皮针" or 题名或关键词:"内皮针"or 摘要:"内皮针" or 主题:"三棱针" or 题名或关键词:"三棱针" or 摘要:"三棱针" or 主题:"梅花针" or 题名或关键词:"梅花针" or 摘要:"梅花针" or 主题:"皮肤针" or 题名或关键词:"皮肤针" or 摘要:"皮肤针" or 主题:"火针" or 题名或关键词:"火针" or 摘要:"火针" or 主题:"干针" or 题名或关键词:"干针"or 摘要:"干针" or 主题:"耳穴贴压" or 题名或关键词:"耳穴贴压"or 摘要:"耳穴贴压" or 主题:"刺络" or 题名或关键词:"刺络"or 摘要:"刺络") and (主题:"随机" or 题名:"随机" or 关键词:"随机" or 摘要:"随机") and (主题:"湿疹" or 题名或关键词:"湿疹"or 摘要:"湿疹" or 主题:"湿疮" or 题名或关键词:"湿疮"or 摘要:"湿疮" or 主题:"浸淫疮" or 题名或关键词:"浸淫疮"or 摘要:"浸淫疮" or 主题:"旋耳疮" or 题名或关键词:"旋耳疮"or 摘要:"旋耳疮" or 主题:"奶癣" or 题名或关键词:"奶癣"or 摘要:"奶癣" or 主题:"乳头风" or 题名或关键词:"乳头风" or 主题:"肾囊风" or 题名或关键词:"肾囊风" or 摘要:"肾囊风")))

Table 6 Search strategy for VIP (Website: http:/lib.eqvip.com)

Search terms
(M=针刺 OR R=针刺 OR M=电针 OR R=电针 OR M=激光针 OR R=激光针 OR M=经皮电刺激神经 OR R=经皮电刺激神经 OR M=经皮电神经刺激 OR R=经皮电神经刺激 OR M=经皮神经电刺激 OR R=经皮神经电刺激 OR M=镇痛皮肤电刺激 OR R=镇痛皮肤电刺激 OR M=经皮电刺激 OR R=经皮电刺激 OR M=TENS OR R=TENS OR M=电止痛 OR R=电止痛 OR M=耳针 OR R=耳针 OR M=耳廓针刺 OR R=耳廓针刺 OR M=头针 OR R=头针 OR M=手针 OR R=手针 OR M=腹针 OR R=腹针 OR M=毫针 OR R=毫针 OR M=内皮针 OR R=内皮针 OR M=三棱针 OR R=三棱针 OR M=梅花针 OR R=梅花针 OR M=皮肤针 OR R=皮肤针 OR M=火针 OR R=火针 OR M=干针 OR R=干针 OR M=耳穴贴压 OR R=耳穴贴压 OR M=刺络 OR R=刺络) AND (M=湿疹 OR R=湿疹 OR M=湿疮 OR

R=湿疮 OR M=浸淫疮 OR R=浸淫疮 OR M=旋耳疮 OR R=旋耳疮 OR M=奶癣 OR R=奶癣 OR M=乳头风 OR R=乳头风 OR M=肾囊风 OR R=肾囊风 OR M=四弯风 OR R=四弯风 OR M=脐疮 OR R=脐疮）AND（M=随机 OR R=随机）

Table 7 Search strategy for CBM (Website: http://www.sinomed.ac.cn/)

Search terms
((("针刺"[常用字段] OR "药物针刺"[常用字段] OR "针刺"[主题词]) OR ("电针"[常用字段] OR "电针"[主题词]) OR "激光针"[常用字段] OR "经皮电刺激神经"[常用字段] OR "经皮电神经刺激"[常用字段] OR "经皮神经电刺激"[常用字段] OR "镇痛皮肤电刺激"[常用字段] OR "经皮电刺激"[常用字段] OR "电止痛"[常用字段] OR "TENS"[常用字段] OR "经皮神经电刺激"[主题词] "OR 耳针"[常用字段] OR ("头针"[常用字段] OR "头针"[主题词]) OR ("手针"[常用字段] OR "手针"[主题词]) OR "腹针"[常用字段] OR ("毫针"[常用字段] OR "毫针"[主题词]) OR "内皮针"[常用字段] OR "三棱针"[常用字段] OR "梅花针"[常用字段] OR "皮肤针"[常用字段] OR "火针"[常用字段] OR "干针"[常用字段] OR ("耳穴贴压"[常用字段] OR "耳穴贴压"[主题词]) OR "刺络"[常用字段]) AND 随机对照试验[文献类型]) AND (((("湿疹"[常用字段:智能] OR "湿疮"[常用字段:智能] OR "浸淫疮"[常用字段:智能] OR "旋耳疮"[常用字段:智能] OR "奶癣"[常用字段:智能] OR "乳头风"[常用字段:智能] OR "肾囊风"[常用字段:智能] OR "四弯风"[常用字段:智能] OR "脐疮"[常用字段:智能])))
